# Web-based platform for analysis of RNA folding from high throughput chemical probing data

**DOI:** 10.1093/nar/gkac435

**Published:** 2022-06-03

**Authors:** Christopher P Jurich, Amir Brivanlou, Silvi Rouskin, Joseph D Yesselman

**Affiliations:** Department of Chemistry, University of Nebraska-Lincoln, Lincoln, NE 68588, USA; Department of Microbiology, Harvard Medical School, Boston, MA 02115, USA; Department of Microbiology, Harvard Medical School, Boston, MA 02115, USA; Department of Chemistry, University of Nebraska-Lincoln, Lincoln, NE 68588, USA

## Abstract

RNA structures play critical roles in regulating gene expression across all domains of life and viruses. Chemical probing methods coupled with massively parallel sequencing have revolutionized the RNA structure field by enabling the assessment of many structures in their native, physiological context. Previously, we developed Dimethyl-Sulfate-based Mutational Profiling and Sequencing (DMS-MaPseq), which uses DMS to label the Watson-Crick face of open and accessible adenine and cytosine bases in the RNA. We used this approach to determine the genome-wide structures of HIV-1 and SARS-CoV-2 in infected cells, which permitted uncovering new biology and identifying therapeutic targets. Due to the simplicity and ease of the experimental procedure, DMS-MaPseq has been adopted by labs worldwide. However, bioinformatic analysis remains a substantial hurdle for labs that often lack the necessary infrastructure and computational expertise. Here we present a modern web-based interface that automates the analysis of chemical probing profiles from raw sequencing files (http://rnadreem.org). The availability of a simple web-based platform for DMS-MaPseq analysis will dramatically expand studies of RNA structure and aid in the design of structure-based therapeutics.

## Graphical Abstract

**Graphical Abstract:**
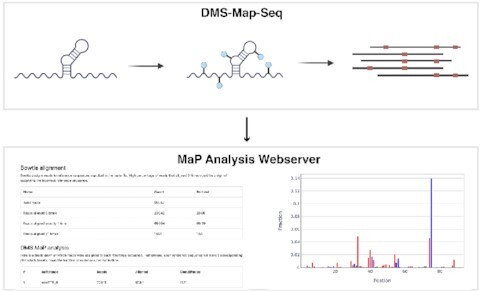
(Top) Cartoon schematic of DMS-MaPseq workflow. (Bottom) The results of this workflow can be analyzed by the MaP analysis server, rendering detailed output and visualizations.

## INTRODUCTION

RNA is an informational intermediate between DNA and proteins and drives a diverse set of biological processes (1). RNA’s ability to execute biological functions is attributable to its ability to form stable secondary and tertiary structures. These RNA structures have been shown to play a critical role in regulating transcription, translation, splicing, protein binding, and cellular localization, among other processes (Reviewed in (2)). Thus, to understand these critical biological processes, we must understand the structure of RNA.

A wealth of techniques have been utilized to study RNA structures at different levels of resolution. Biophysical approaches such as NMR and X-ray crystallography have proven useful at resolving RNA structures at an atomic scale and can provide information on tertiary structure; however, these high-resolution approaches are time-intensive, requiring pure samples, making them not ideal for high-throughput data collection. In contrast, when combined with next-generation sequencing, chemical probing has allowed for the elucidation of the secondary structure of RNAs in a high throughput manner both in vivo and in vitro with single-nucleotide precision (3–6). One such chemical probing method is Dimethyl-Sulfate-based Mutational Profiling and Sequencing (DMS-MaPseq) (7), which utilizes Dimethyl-Sulfate (DMS) to methylate the Watson-crick face of unpaired adenines and cytosines in an RNA molecule.

DMS-MaPseq has been used to determine the genome structure of both HIV-1 (8) and SARS-CoV-2 in vivo (9), which gives insight into the fundamental biology of these viruses and leads to the development of novel therapeutics (10). DMS-MaPseq has also been used to show how RNA structures contribute to the regulation of translation (11,12) and cold shock adaptation in bacteria (13). Due to its relatively simple experimental setup, DMS-MaPseq continues to be a powerful tool in elucidating RNA structures in vivo and in vitro.

The high-throughput nature of DMS-MaPseq produces orders of magnitude more data than previous chemical mapping procedures leading to a new analysis bottleneck. Furthermore, incorporating next-generation sequencing requires a host of new bioinformatics tools that may be unfamiliar to RNA structural biologists. These tools also require new computational infrastructure and know-how to run. These new challenges can lead to a significant barrier to analyzing high-throughput chemical mapping data. We have developed a MaP analysis webserver to alleviate the computational burden for extracting RNA structure information from mutational profiling chemical mapping data. This web-based platform will enable the scientific community to analyze high throughput chemical probing data without dedicated computational knowledge or infrastructure.

## METHOD OVERVIEW

The DMS-MaPseq protocol is extensively detailed in a dedicated paper (see ref: (7)). In short, RNA is modified with DMS which methylates the N1 position of solvent accessible Adenosines (As) and the N3 position Cytosines (Cs). Modified RNA is then reverse transcribed using a thermostable group II reverse transcriptase (TGIRT), which produces mutations on the cDNA at corresponding modified As and Cs. The resulting cDNAs are sequenced and aligned to a reference sequence to calculate the frequency of mutations at individual bases. As and Cs with high mutational frequency are likely unpaired as their Watson-Crick face was accessible to methylation by DMS. For example, a mutational fraction of 0.1 indicates that 10% of the sequenced reads had a mutation at the corresponding position. The mutational profile of the RNA molecule can be used to determine the secondary structure of an RNA as well as specific tertiary structure features.

## WEBSERVER

### Webserver submission

**Figure 1.**
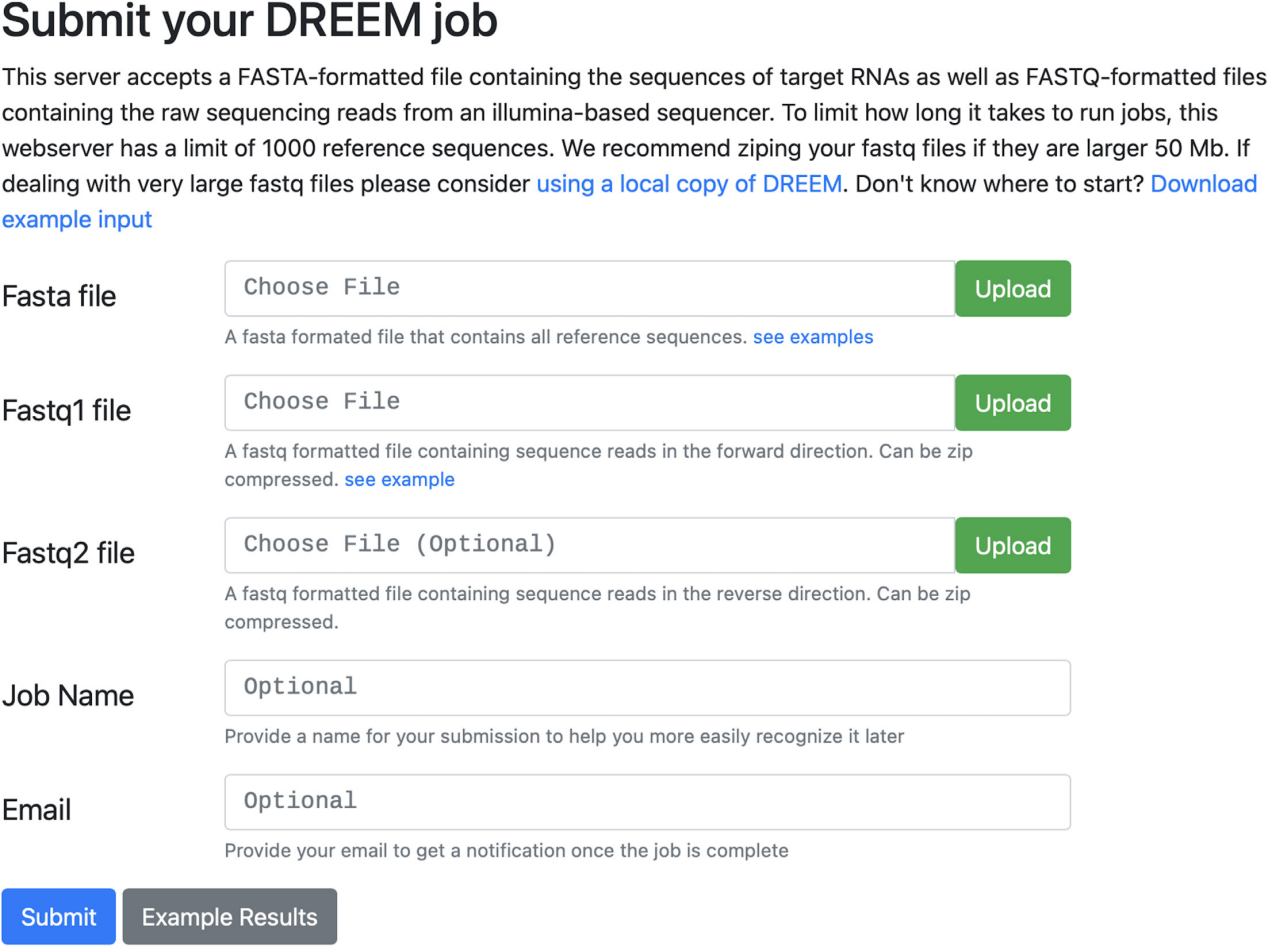
Submission page. The submission page is designed to be lightweight and accessible. The only two required inputs are a FASTA-formatted file containing the reference sequences and a FASTQ-formatted file containing the sequencing reads from an Illumina-based sequencer. There are also optional arguments for submitting a second FASTQ file if the reads are paired, a job name for self-reference, and an email address to be alerted when the job finishes.

The MaP analysis server uses the lightweight CherryPy (https://cherrypy.dev) framework based on Python3 (https://www.python.org/) for server web services and data management. The client-side utilizes HTML5 (http://www.w3.org/TR/html5/) styled with Bootstrap (http://getbootstrap.com/) coupled with jQuery (http://jquery.com) for interactive user-interface components. The MaP analysis server supports most web browsers, including Google Chrome, Mozilla Firefox, Apple Safari, and Microsoft Edge. The job submission page has been streamlined to be user-friendly (Figure 1). Submitting a job requires only two file inputs. (1) A FASTA file containing the probed RNA sequences with their corresponding names. (2) A single FASTQ file containing the results of an Illumina sequencer containing forward reads with sequences with corresponding quality scores. If sequencing was pair-ended, one could also supply the reverse reads in the Fastq2 file input. The server also accepts zip compressed files which reduce upload times by over 90%. Jobs can be named for convenience. Furthermore, a valid email address can be supplied, which will alert the user by email upon job completion.

### Results page

**Figure 2.**
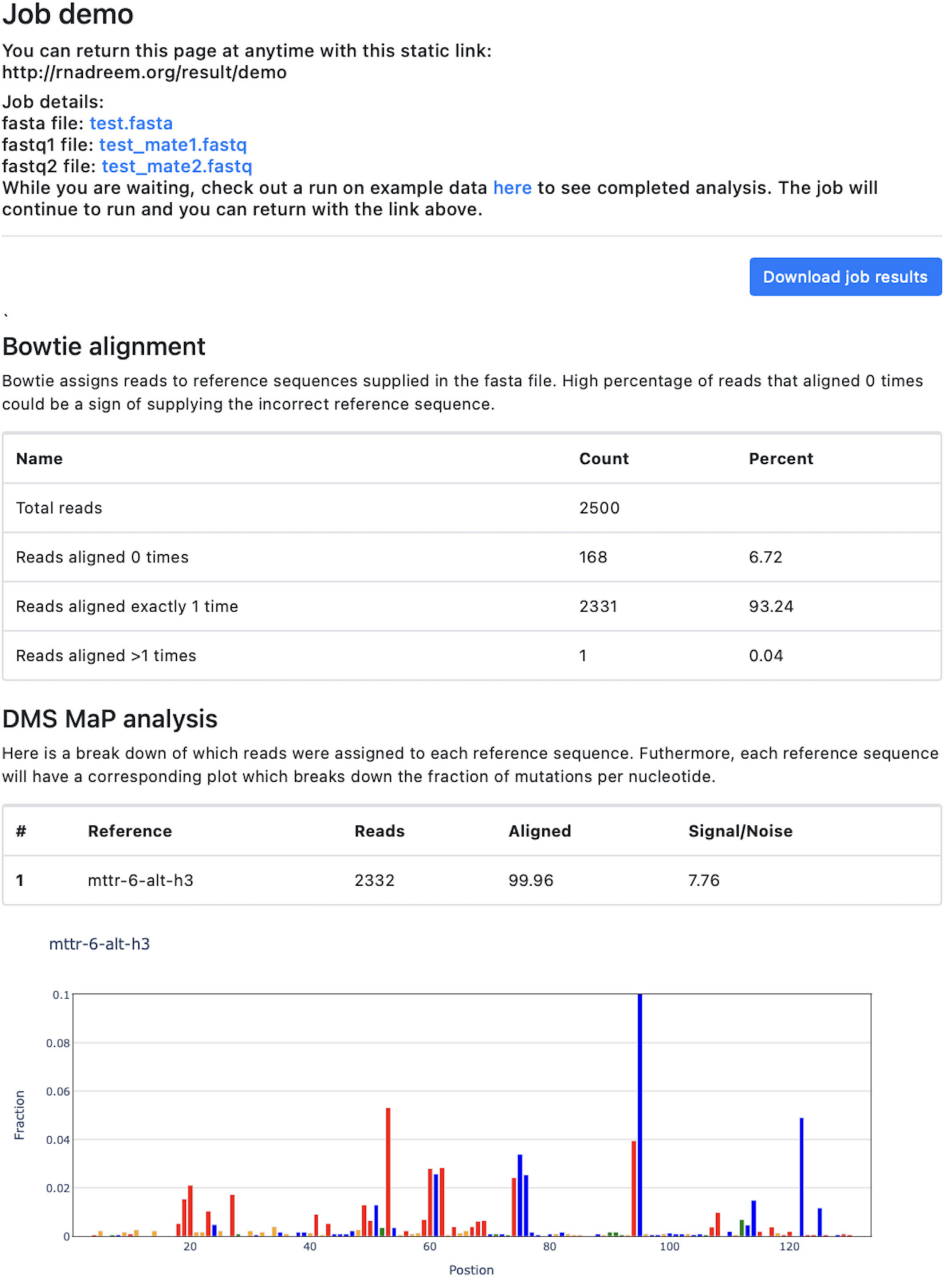
Results page. The results page gives a detailed breakdown of the results of the MaP analysis. (Top) A summary of job information, including links to download files submitted to the sever and a button to download all results from the job as a zip file. (Middle) A table summarizing the sequence alignment by bowtie2. (Bottom) A summary of the mutational fraction per nucleotide in graphical form. Nucleotides are colored as guanosine (orange), cytidine (blue), adenosine (red), and uridine (green).

Upon submission, each job is generated with an ID assigned to a static link to the results page in the form http://rnadreem.org/result/JOB_ID which can be accessed for up to three months. The results are parsed by Python and rendered onto the web page, as shown in Figure 2. At the top of the results page are links to download the supplied FASTA and FASTQ files. There is also a link to download a zip of all the job results under ‘Download job results.’ The main body of the output page is broken up into two sections. In the first ‘Bowtie alignment,’ there is a detailed breakdown of the sequence alignment for all reference DNAs with the bowtie2 aligner (14). The table summarizes ‘Total reads’ – the number of reads found in the supplied FASTQ files, ‘Reads aligned 0 times’ – reads that did not align to any of the reference sequences, ‘Reads aligned exactly 1 time’ – reads that aligned to one sequence in one position, and ‘Reads aligned > 1 times’ – reads that aligned to multiple reference sequence or different positions on the same sequence. Users should ensure that few sequences align 0 times while most sequences align at least 1 time. Aligning more than one time is not necessarily an issue. The second part of the analysis will only accept reads that align significantly better to one reference sequence over another.

The second section, ‘DMS Map analysis,’ details the mutation fraction for each nucleotide in a reference sequence. These mutation fractions are directly proportional to the DMS modification rate at a given nucleotide position. First, a table summarizes the total number of reads, the alignment rate, and signal to noise for each reference sequence. This alignment rate reports the percentage of reads removed based on stricter criteria than the initial bowtie2 alignment. This alignment should be relatively high (> 90%), and if it's lower, this may be a sign of an incorrect reference sequence or too many similar reference sequences. For each reference sequence, an interactive bar plot displays the mutational fraction of each nucleotide. Coloring is static, with guanosine, cytidine, adenosine, and uridine as orange, blue, red, and green. These interactive plots were generated with Plotly (https://plotly.com) and support various functions, such as hovering for more information and zooming. Investigating the mutation fraction per nucleotide can reveal structural details for a reference RNA sequence. Generally, As and Cs with low reactivity (mutation fraction < 0.01) are in Watson-crick base pairs (e.g. AU or GC pairs). Conversely, nucleotides with high reactivity are unpaired or in noncanonical interactions. For a more in-depth discussion of DMS reactivity values, please see refs: (7–9).

### Webserver use cases

**Figure 3.**
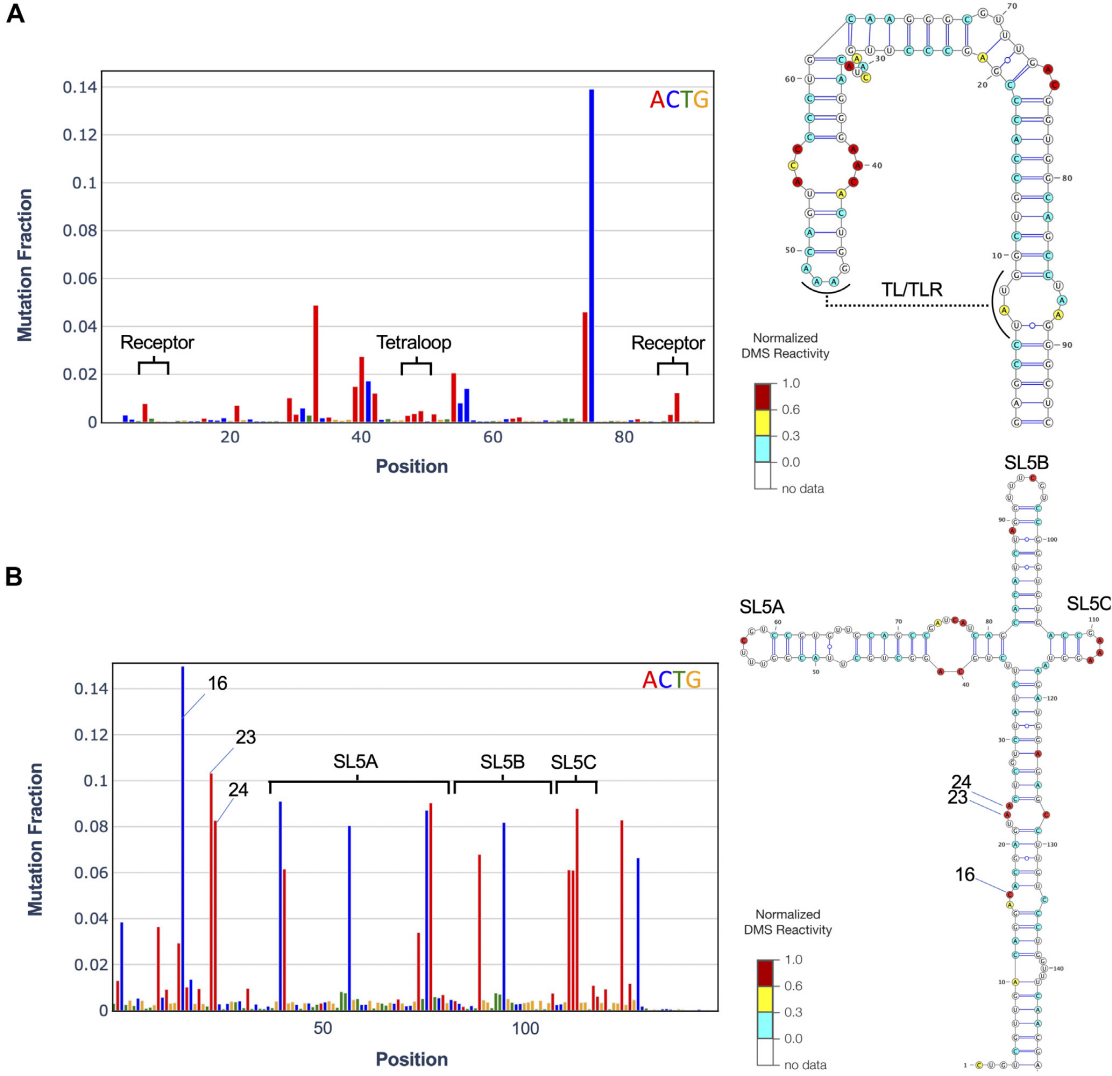
RNA structure information derived from MaP analysis webserver. DMS-MaPseq raw data and structure models for (**A**) Rationally designed RNA nanostructure with a TL/TLR tertiary contact. This contact comprises a tetraloop and receptor, which have significantly reduced DMS reactivity, as highlighted on the left panel. (**B**) Stem-loop 5 (SL5) in SARS-CoV-2. DMS signal and structure model highlighting a few of the most reactive bases (nts 16, 23,24).

To demonstrate the versatility of the MaP analysis server, we have selected two different use cases. First, in the design of RNA nanostructures that rely on rationally designed tertiary contacts, DMS can be a powerful probe for their formation. In our example, we utilized a tetraloop/tetraloop loop receptor (TL/TLR) (15,16). This strong tertiary contact contains three As on the tetraloop and one A on the receptor involved in the interaction. These become protected from DMS modification upon formation of the tertiary contact (Figure 3A). DMS thus represents a rapid probe for 3D structure features and can be used for screening constructs before utilizing rationally constructed nanostructures for downstream applications. As a second example, we show the DMS reactivity can derive a secondary structure model of Stem Loop 5 (SL5) in the 5′ untranslated region of SARS-CoV-2 (Figure 3B). The secondary structure of SL5 has been validated extensively using homology modeling, SHAPE-MaP, icSHAPE, RNase and inline probing, and NMR. The DMS-MaPseq driven model gives ∼99% agreement with literature consensus structure (9). This example demonstrates how DMS reactivities can be used as constraints in computational prediction to generate accurate secondary structure models.

## CONCLUDING REMARKS

In the past decade, the coupling of next-generation sequencing to chemical mapping has seen the emergence of rapid and high-throughput probing experiments. We expect experiments such as DMS-MaPseq to increase in popularity, with more groups adopting and expanding chemical mapping to supplement their other molecular and structural biology experiments. With the ever-increasing usage of high-throughput chemical mapping techniques, there will be a corresponding demand for new computational resources to analyze the results of these experiments. The webserver presented herein brings the first free and easy-to-use computational tool for analyzing MaP chemical mapping experiments. The MaP analysis webserver will alleviate the existing computational burden allowing to dramatically expand studies of RNA structure and aid in the design of structure-based therapeutics.
